# Turkish validity and reliability study on the quick aphasia battery

**DOI:** 10.1002/brb3.3343

**Published:** 2023-12-31

**Authors:** Mümüne Merve Parlak, Ayşen Köse

**Affiliations:** ^1^ Department of Speech and Language Therapy, Faculty of Health Sciences Ankara Yıldırım Beyazıt University Ankara Turkey; ^2^ Department of Speech and Language Therapy, Faculty of Health Sciences Hacettepe University Ankara Turkey

**Keywords:** aphasia, language, quick aphasia battery, quick, Turkish, validity and reliability

## Abstract

**Introduction:**

The quick aphasia battery (QAB), which assesses all areas of language in detail and quickly, was developed in English. It has been shown to be suitable for bedside patients. There is a need for a Turkish bedside test that allows for a comprehensive yet rapid assessment of stroke patients in terms of aphasia. The aim of this study was to create a Turkish version of QAB (QAB‐TR) and to determine its validity and reliability in Turkish‐speaking patients after a stroke.

**Materials and methods:**

The study was conducted with 188 people aged 41–88 years. Of these, 37 (19.7%) had aphasia (12 chronic, 25 acute), 53 (28.2%) were acute stroke patients without aphasia, and 98 (52.1%) were healthy controls. Internal consistency and criterion validity, test–retest reliability, and inter‐rater reliability of the QAB‐TR were performed. The language assessment test for aphasia was used for criterion validity. For the inter‐rater reliability of the test, two different speech language therapists (SLP) administered the QAB‐TR. For test–retest reliability, 2 weeks later, the same SLP who filled out the QAB‐TR the first time was administered the test again. To test the validity of the test, correlations between the items and subsections were determined. Receiver operating characteristic (ROC) analysis was performed to examine the sensitivity and selectivity of the QAB‐TR score, and a cut‐off value was determined to distinguish patients with aphasia.

**Results:**

The inter‐rater Krippendorff's alpha value of the QAB‐TR total was 0.6754. There was no statistically significant difference (*p* > .05) between the first and second QAB‐TR total scores. The correlation analysis between the QAB‐TR subsection scores and the total QAB‐TR score (0.244–0.897) revealed statistically significant relationships. The area under the ROC curve was statistically significant and was found to be 0.853 (95% confidence interval: 0.799–0.906). The cut‐off point for the QAB score to discriminate between patients with aphasia and those without aphasia was found to be 8.825, with 0.767 sensitivity and 0.765 selectivity (1–0.235).

**Conclusion:**

All the study results show that QAB‐TR has internal consistency, criterion validity, test–retest reliability, and inter‐rater reliability. It can be administered in as little as 15 min and provides information about the multidimensional linguistic profiles of individuals. QAB‐TR can be used for both clinical and study purposes as a language battery that allows for the measurement of the strengths and weaknesses of Turkish‐speaking individuals who have suffered a stroke in basic language areas in acute and chronic periods. It can be easily administered at the bedside for individuals who have just suffered an acute stroke and can facilitate early assessment of individuals in terms of aphasia and early initiation of therapy, if necessary.

## Graphical abstract

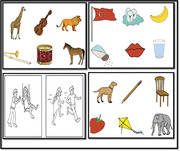

## LIMITATIONS

In the present study, the second and third forms of QAB‐TR were administered to 10 individuals. The second and third forms were not administered to individuals who had just had an acute stroke. Therefore, the cut‐off scores of the second and third forms were not determined. This constitutes the main limitation of the present study.

## 1 INTRODUCTION

Stroke is defined as a temporary or permanent disruption of blood flow to the brain and the symptoms it causes (Hallowell, 2023; Parlak et al., 2022). It is also the leading cause of death worldwide, after heart attacks (World Health Organization, 2020). Stroke and stroke‐related disorders affect various brain areas and are the most common causes of aphasia (American Speech‐Language‐Hearing Association, n.d.; Roth & Worthington, 2019; Toğram & Maviş, 2012).

Aphasia, in its most general definition, is an acquired language disorder characterized by various aspects of the language system due to brain damage (Sheppard & Sebastian, 2021). The prevalence of aphasia varies depending on the population studied and the specific causes of aphasia. In stroke patients, it is reported to vary between 19% and 62% (Flowers et al., 2013; Kadojić et al., 2012). A communication disorder was identified in 64% of 88,974 stroke patients in a recent study. A total of 12% of the individuals affected only had aphasia, whereas 28% had both dysarthria and aphasia (Mitchell et al., 2023).

Speech and language therapists (SLPs) should conduct a detailed language assessment to determine the degree of impairment in the language areas caused by stroke and to reduce its effects (American Speech‐Language‐Hearing Association, n.d.). Aphasia is multidimensional, so it is thought that aphasia assessment should reflect the neural and functional mechanisms that are impaired and preserved. The aim of an aphasia assessment test is to first determine whether aphasia is present, and then, if aphasia is present, to provide detailed information about the nature of the condition (American Speech‐Language‐Hearing Association, n.d.; Spreen & Risser, 2003; Toğram & Maviş, 2012).

Although many tests assess one or more aspects of language disorders in people with aphasia (PWA), there are relatively few sufficiently standardized tests. Moreover, batteries, such as the Boston Aphasia Examination and the Western Aphasia Battery, may take more than 45 min to administer (Goodglass & Kaplan, 1983; Kavakci et al., 2022; Shewan & Kertesz, 1980). Although abbreviated versions of some aphasia batteries are available, the lack of standardization and other difficulties associated with their use make them suboptimal for use in this population (Kavakci et al., 2022; Toğram & Maviş, 2012). In order to assess fluent speech, different dimensions, such as word finding, grammatical construction, and motor speech, should be evaluated. However, the lack of an aphasia assessment battery that meets these criteria in the literature. In this context, the quick aphasia battery (QAB), which assesses all areas of language in detail and quickly, was developed by Wilson et al. (2018).

QAB is a rapid and easy‐to‐administer aphasia test developed in English, and validity and reliability studies have been conducted on it (Wilson et al., 2018). It has been shown to be suitable for bedside use and has been successfully translated in other languages, including Portuguese and French. In addition, it has been administered for an average of 18.9 ± 7.3 min in PWA, including chronic PWA, and for an average of 11.6 ± 3.0 min in PWA (Wilson et al., 2018). QAB includes eight subtests: (1) consciousness level; (2) connected speech; (3) word comprehension; (4) sentence comprehension; (5) picture naming; (6) repetition; (7) reading aloud; and (8) motor speech. Each subtest contains 5–12 items, each scored based on a scale ranging from 0 to 4. However, for the parameters evaluated in general, 0 indicates the worst level and 4 indicates the best level. The precise meaning of each score on the scale varies by item and is indicated on the assessment form. QAB has three separate assessment forms and its own Excel scoring system, based on which the eight subdomains assessed in the form can be scored. The score summary provides a profile of the test taker's preserved and impaired language areas (Wilson et al., 2018).

In Turkey, there are two widely used Turkish tests for the assessment of PWA: (1) the Gülhane Aphasia Test‐2 and (2) the language assessment test for aphasia (LATA) (Maviş et al., 2007; Toğram & Maviş, 2012). In the Turkish validity and reliability tests, there is no section that evaluates connected speech with scores during both spontaneous speech and picture description. In addition, as the LATA is a comprehensive test, its administration time reaches up to 40 min in PWA. This makes it difficult to apply, especially in patients in the acute bedside period. An inter‐rater reliability study of the Aphasia Rapid Test in Turkish has been conducted, but its validity and test–retest reliability have not been established (Kavakci et al., 2022). It is also reported that although this test is effective in determining aphasia, it provides insufficient information about the strengths and weaknesses of the language skills of PWA (Wilson et al., 2018). Thus, there is a need for a bedside test that allows for a comprehensive yet rapid assessment of stroke patients in terms of aphasia. QAB is a new aphasia battery that can be administered to PWA in a short period and provides multidimensional profiles of individual patients by measuring their strengths and weaknesses in basic language domains. Therefore, the primary aim of this study was to create a Turkish version of the QAB (QAB‐TR) and to determine the validity and reliability of the QAB‐TR for aphasia detection. The secondary aim of the study was to examine the usability of the QAB‐TR for assessing the language skills of Turkish‐speaking patients after stroke.

## 2 METHODS

The present study was performed at the Neurology Clinic of the University of Health Sciences, Dışkapı Training and Research Hospital, with the approval of the ethics committee (Approval no. 139/25).

### 2.1 Procedure

The present study consisted of two steps. The first step involved the translation and adaptation of QAB into Turkish, and the second step involved testing the validity and reliability of the adapted test.

The QAB was adapted into Turkish by the researchers after obtaining permission from its responsible author. The word comprehension, picture‐naming, repetition, and reading‐aloud sections of QAB‐TR were adapted to Turkish culture in accordance with the QAB development steps. Pictures that were not appropriate for Turkish culture were removed, and new pictures were drawn by a graphic designer. The same procedure was applied to all the three forms of QAB‐TR (QAB‐TR Form 1, QAB‐TR Form 2, and QAB‐TR Form 3). The word comprehension section of QAB‐TR requires phonologically related words. For this reason, pictures of phonologically similar words in Turkish were drawn. In all forms of QAB‐TR (Form 1, Form 2, and Form 3), the second part of the word comprehension section was organized according to Turkish phonological similarity. For example, in QAB, the phonological related word for “boot” was “boat,” whereas in QAB‐TR, the phonological related word for “buz” was “tuz, muz.”

The adapted version of QAB‐TR was sent to five speech‐language pathologists (with at least 3 years of experience in this field of aphasia), and their opinions on the appropriateness of the items and their translations were obtained. Expert opinions were obtained through face‐to‐face interviews by opening the form from the responsible author's computer. In the meantime, the comprehensibility of all items and the appropriateness of the pictures were asked. The questions that each expert did not find comprehensible were noted, and the items that at least three of the five experts did not find appropriate were modified. The revised version of these items was asked again to the experts who found them inappropriate, and the pilot study was started after approval was obtained. A pilot study was conducted with 18 healthy individuals, and the comprehensibility of the items was checked.

**FIGURE 1:**
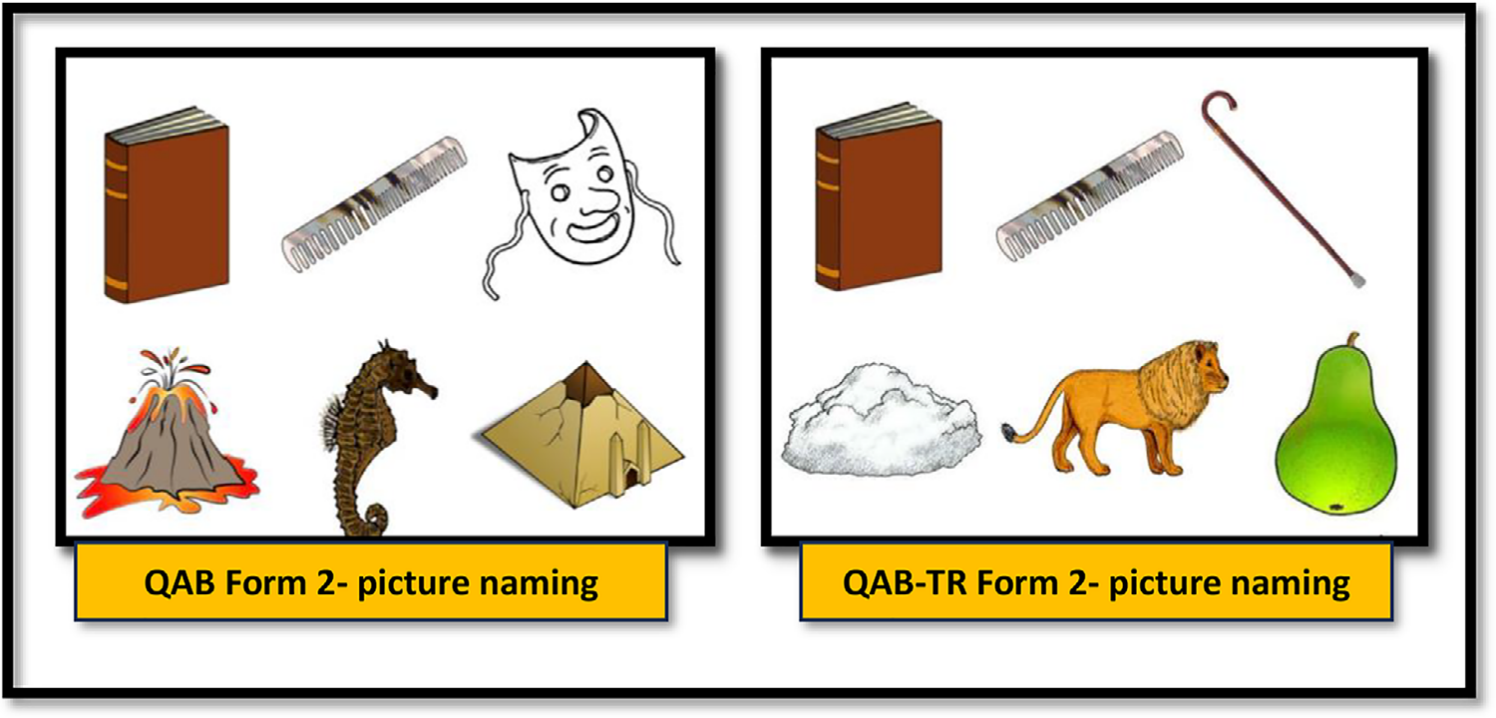
Changes to the picture naming section of Form 2 in the Turkish version of QAB (QAB‐TR) adaptation.

Some changes were made according to the expert opinions and the results of the pilot study. In the first semantically related part of the word comprehension section, no changes were made to the pictures in QAB‐TR Form 1. However, changes were made to semantically related words in Form 2 and Form 3. For example, both harp and saxophone in Form 2 could not be answered by healthy individuals in the pilot study because they are not well known in Turkish culture. For this reason, the harp was removed and replaced with a picture of a piano. The picture‐naming section was organized in all forms. Unfamiliar pictures that could not be named by many people, even if they had a high level of education, were removed from the picture‐naming section. Instead of these pictures, the pictures used in the phonetic similarity vocabulary comprehension section of the original QAB or the new pictures that were drawn were used. For example, in Form 2, volcano, seahorse, mask, and pyramid were replaced with cloud, lion, cane, and pear (Figure 1). The sentence comprehension section was organized because it was observed that sentences with passive verbs were difficult to understand. For example: “Do mothers take care of the babies? Bebeklere anneleri mi bakar”; “Do mice chase cats? Fareler kedileri kovalar mı?” passive sentences were changed to active constructions instead of “Are babies watched by babysitters? Bebekler bakıcılar tarafından izleniyor mu?”, “Are cats chased by mice? Kediler fareler tarafından mı kovalanıyor?”

After the scale was edited, it was back‐translated into English by a translator who knew both Turkish and English but was not familiar with QAB‐TR. After the wording of QAB‐TR's English retranslation was found to be quite similar to that of the original English scale, QAB‐TR was administered to the participants of the present study, who had been selected based on the inclusion and exclusion criteria. The assessment tests were administered bedside to stroke patients with or without aphasia in the acute phase while they were hospitalized in the neurology clinic. The assessments were videotaped for patients who consented to be videotaped during administration. Patients with chronic stroke were screened from the system records, and healthy people were identified through snowball sampling, contacted by phone, informed about the study, and asked to present themselves for evaluation in the outpatient clinic room based on the inclusion and exclusion criteria.

The valid and reliable LATA was also administered to all the participants to assess the criterion validity of QAB‐TR. As LATA takes a long time to administer to inpatients, the first LATA was administered in the morning. QAB‐TR Form 1 was administered when the inpatients had rested for 1 h to prevent fatigue. To determine the test–retest reliability of QAB‐TR Form 1, it was administered to some healthy and chronic aphasic individuals and was administered again to 42 individuals after 2 weeks. To determine the scale's inter‐rater reliability, the evaluation videos (Form 1) of 30 individuals who had just suffered an acute stroke were scored by another speech‐language pathologist student who had been trained in applying and evaluating the test and who did not know the initial evaluation results. In addition, three separate forms of QAB‐TR were administered to 10 healthy individuals. Statistical analyses were conducted to determine a cut‐off score to discriminate aphasia and the test's repeated validity, inter‐rater reliability, and criterion validity for the Turkish population.

### 2.2 Participants

In the present study, QAB‐TR was administered to three groups of participants: (1) acute and chronic stroke patients with aphasia, (2) acute stroke patients without aphasia, and (3) healthy controls. Patients with acute stroke were hospitalized in the neurology clinic of the hospital. Patients with chronic stroke were screened from the hospital system, contacted by phone, and invited to participate in the study. Healthy people were identified through snowball sampling and invited to participate in the study.

For the first and second groups (i.e., all stroke patients), the inclusion criteria were as follows: having volunteered to participate in the study, having Turkish as the mother tongue, being fluent in Turkish before the stroke, being literate, having an acute‐phase condition stable enough to take the tests, and having been confirmed by computed tomography or diffusion‐weighted magnetic resonance imaging to have had a stroke. The exclusion criteria for stroke patients were having an impaired cognitive or language function due to dementia or any other reason and having a major psychiatric disorder. A previous stroke was not included in the exclusion criteria unless there was ongoing cognitive or language impairment during the most recent stroke.

For the healthy controls, the inclusion criteria were having volunteered to participate in the study; being literate; having no major psychiatric disorder, stroke, brain injury, or neurological diagnosis, such as Parkinson's disease or dementia; and having a Standardized Mini‐Mental State Examination (SMMSE) score between 27 and 30.

The study was conducted with 188 people aged 41–88 years who met the inclusion and exclusion criteria. Of these, 37 (19.7%) had aphasia (12 chronic and 25 acute), 53 (28.2%) were acute stroke patients without aphasia, and 98 (52.1%) were healthy controls. The mean age of the participants was 64.26 ± 10.213, and 16 (8.5%) were literate, 82 (43.6%) were primary school graduates, 25 (13.3%) were secondary school graduates, 36 (19.1%) were high school graduates, and 29 (15.4%) were university graduates. Details of the participants are presented in Table 1.

**TABLE 1:** Demographic characteristics of the participants.

	All participants	Healthy control participants	Acute and chronic stroke patients with aphasia	Acute stroke patients without aphasia
**Number of participants**	188	98	37	53
**Age (years)**	64.26 ± 10.213	61.93 ± 9.226	71.03 ± 8.896	63.83 ± 10.887
**Sex (M/F)**	93/95	47/51	15/22	31/22
**Handedness (R/L)**	179/9	93/5	35/2	51/2
**Education (years)**	8.01 ± 4.736	9.50 ± 4.377	4.32 ± 3944	7.83 ± 4.501
**Days post stroke (acute/chronic)**	–	–	4.00 ± 1.333/743.75 ± 228.921	4.26 ± 1.041

### 2.3 Assessment tools

#### 2.3.1 Language assessment test for aphasia

Toğram and Maviş (2012) examined the validity and reliability of the test. LATA consists of eight subtests assessing speech fluency, auditory comprehension, repetition, naming, reading, speech acts, grammar, and writing. Its total score is 292 points. An increase in the LATA total score indicates that individuals are less affected in terms of aphasia. For the diagnosis of aphasia, a separate cut‐off score is scored according to the level of education for the age groups of 23–59, 60–74, 75, and over. It is thought that the severity of aphasia increases as the score decreases. However, an aphasia classification, such as mild, moderate, or severe aphasia, cannot be made for individuals who are found to have aphasia (Toğram & Maviş, 2012).

#### 2.3.2 Standardized mini‐mental state examination

To determine whether the cognitive levels of the individuals after the stroke and the healthy cognitive levels were sufficient, the SMMSE, whose validity and reliability for the Turkish population had been established by Güngen et al. (2002).

#### 2.3.3 Quick aphasia battery‐Turkish

**FIGURE 2:**
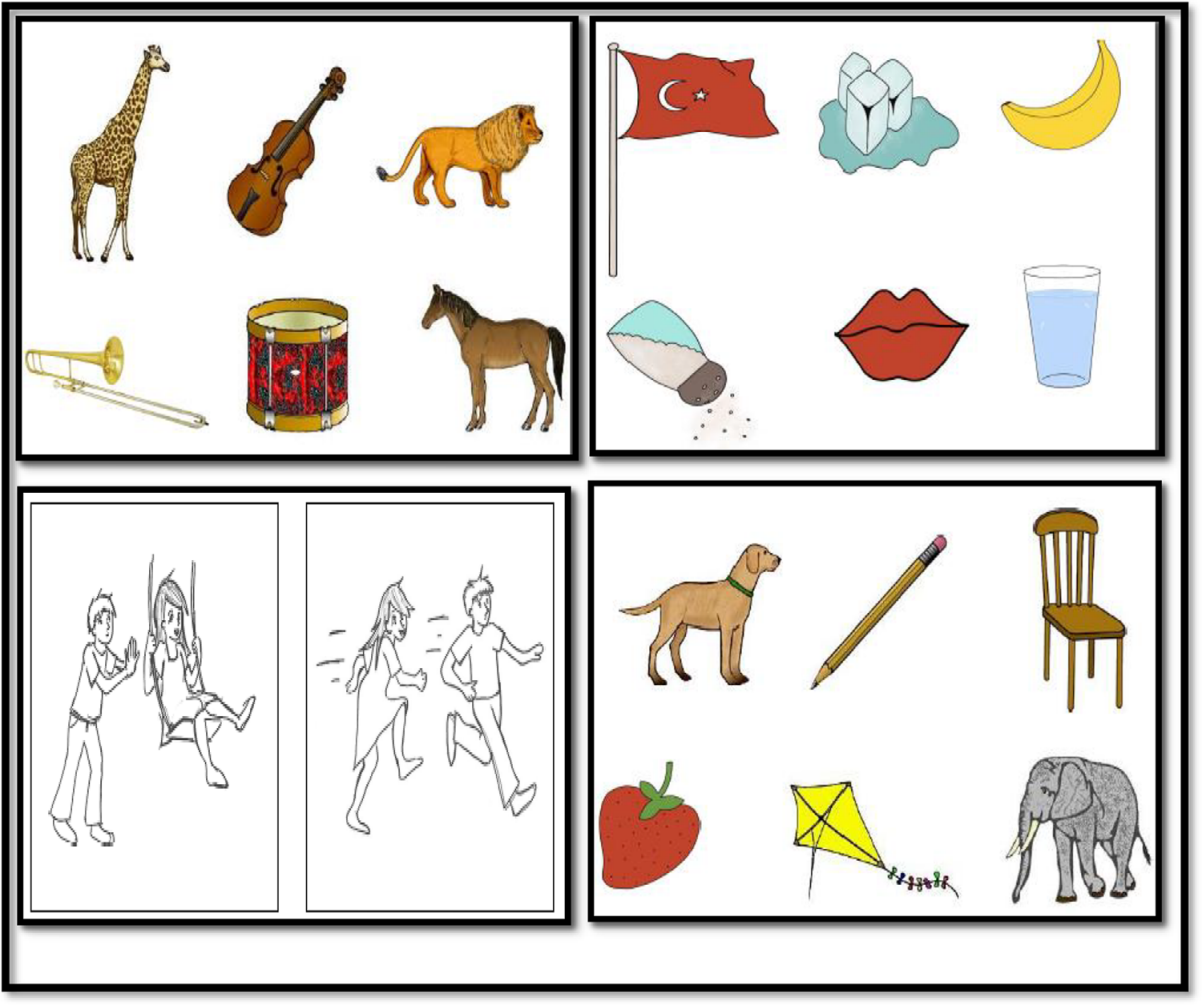
Some subsection pictures in Turkish version of QAB (QAB‐TR) Form 1.

QAB‐TR has eight subsections: level of awareness, word comprehension, sentence comprehension, word finding, grammar, motor speech, repetition, and reading. Some subsection pictures in QAB‐TR Form 1 are shown in Figure 2.

For the QAB‐TR subsections and total score, an Excel scoring form based on the scoring system of the original QAB was used (Wilson et al., 2018). The scores for word comprehension, sentence comprehension, word finding, grammar, motor speaking, repetition, and reading were converted, and a total QAB score of over 10 points was obtained. All summary measures were out of 10 and were calculated by dividing the resulting score by the denominator and then multiplying by 10, or the appropriate percentage of 10, as indicated. Word finding consisted of a picture naming score (60%) and connected speech scores for word finding (40%). The grammatical structure measure was 80% derived from connected speech measures. Agrammatism contributes 40% of the score, whereas reduced length and sentence complexity contribute 20%. Paragrammatism contributes 20% but is limited to not exceeding the agrammatism score. The remaining 20% of the summary measure of grammatical structure reflects scores for sentence elements in the repetition and reading subtests. The details of the percentages used in the Excel score calculation for QAB‐TR subsections and QAB‐TR total are shown in Table 2.

**TABLE 2:** Calculation of summary measures (Wilson et al., 2018).

Summary measure	Definition	
**Word comprehension**	Word comprehension total, corrected for chance by subtracting 8 and clipping at 0; denominator is now 24	
**Sentence comprehension**	Sentence comprehension total, corrected for chance by subtracting 24 and clipping at 0; denominator is now 24	
**Word finding**	60%	Picture naming total
	20%	Connected speech: anomia
	20%	Average of connected speech: empty speech, semantic paraphasias, and phonemic paraphasias, but capped so as not to exceed anomia
**Grammatical construction**	40%	Connected speech: agrammatism
	20%	Connected speech: Reduced length and complexity
	20%	Connected speech: Paragrammatism, but capped so as not to exceed agrammatism
	20%	Average of sentence items from repetition and reading subtests
**Speech motor programming**	Motor speech: apraxia of speech	
**Repetition**	Repetition total	
**Reading**	Reading aloud total	
**QAB‐TR overall**	18%	Word comprehension summary measure
	18%	Sentence comprehension summary measure
	14%	Word finding summary measure
	14%	Grammatical construction summary measure
	8%	Speech motor programming summary measure
	8%	Repetition summary measure
	8%	Reading summary measure
	8%	Connected speech: overall communication impairment
	2%	Connected speech: reduced words per minute
	2%	Connected speech: self‐correction

### 2.4 Statistical analysis

The research data were analyzed using the SPSS 26 program. The descriptive findings are presented herein as number, percentage, mean, and standard deviation values. The agreement between the two different raters was evaluated via Krippendorff's alpha analysis (Krippendorff, 1995). The normality assumption of the variables was evaluated by considering their kurtosis and skewness values. Kurtosis and skewness values within the range of ± 1.5 indicated normal distribution (Tabach et al., 2013). Nonparametric tests were used when the sample sizes of the groups to be compared were less than 30.

Analysis of variance and the Kruskal–Wallis test were used to compare the three independent groups, and when significant differences were found, the Bonferroni multiple‐comparison correction test was used to determine the groups with differences. The differences between the two paired measurements were analyzed using the Wilcoxon signed‐rank test.

The differences between the three paired measurements were evaluated using the Friedman test. Spearman's correlation coefficient was used in the variables’ correlation analyses. Correlation coefficients in the range of ±0.70 to 1.00 were interpreted as indicating the existence of high‐level relationships between the variables; ±0.70 to 0.30, medium‐level relationships; and ±0.30 to 0.00, low‐level relationships (Gürbüz, 2019).

Receiver operating characteristic (ROC) analysis was performed to examine the sensitivity and selectivity of the QAB‐TR (Form 1) score, and a cut‐off value was determined to distinguish patients with aphasia (Karagöz, 2016). A *p*‐value < .05 was considered significant in the evaluation of the analysis results.

## 3 RESULTS

The inter‐rater Krippendorff's alpha value of QAB‐TR total when the results of 30 individuals were reevaluated by a second rater was 0.6754.

There was no statistically significant difference (*p* > .05) between the first QAB‐TR total score (9.52 ± 0.614) and the second QAB‐TR total score (9.46 ± 0.732) (Table 3). In the Spearman correlation test between the first and second total scores, it was found that they had a highly significant positive correlation (*r* = .843).

**TABLE 3:** Results of the difference analysis between the first and second Turkish version of QAB (QAB‐TR) measurements.

		*n*	Mean	Standard deviation	*Z/t*	*p*
Word comprehension	First measurement	42	9.73	0.595	−1.131	.258
	Second measurement	42	9.59	1.010		
Sentence comprehension	First measurement	42	8.49	2.110	−0.472^a^	.639
	Second measurement	42	8.60	2.001		
Word finding	First measurement	42	9.81	0.389	−0.462	.644
	Second measurement	42	9.72	0.735		
Grammatical construction	First measurement	42	9.75	0.709	−1.454	.146
	Second measurement	42	9.49	1.139		
Speech motor programming	First measurement	42	9.64	1.303	−1.000	.317
	Second measurement	42	9.70	1.131		
Repetition	First measurement	42	9.84	0.485	−1.378	.168
	Second measurement	42	9.94	0.235		
Reading	First measurement	42	9.68	0.806	−2.094	**.036**
	Second measurement	42	9.89	0.356		
QAB‐TR total	First measurement	42	9.52	0.614	−0.226	.821
	Second measurement	42	9.46	0.732		

^a^ *t* test.

It was also determined that there was a positive and highly significant correlation (*r* = .812) between the total LATA score and the total QAB‐TR score. In addition, a statistically significant medium–high correlation was found between the LATA subsections and the similar QAB‐TR subsections (Table 4).

**TABLE 4:** Correlation results between language assessment test for aphasia (LATA) subsections and similar Turkish version of QAB (QAB‐TR) subsections.

LATA	QAB	*r*	*p*
Comprehension of objects	**Word comprehension**	*r* = .607	.000
Auditory comprehension total	**Word comprehension**	*r* = .595	.000
Auditory comprehension total	**Sentence comprehension**	*r* = .458	.000
Comprehension of Yes/No questions	**Sentence comprehension**	*r* = .441	.000
Grammar	**Grammatical construction**	*r* = .731	.000
Word actions	**Grammatical construction**	*r* = .616	.000
Speech fluency	**Grammatical construction**	*r* = .719	.000
Naming by looking at the picture	**Word finding**	*r* = .716	.000
Naming	**Word finding**	*r* = .756	.000
Word reading	**Reading**	*r* = .662	.000
Reading total	**Reading**	*r* = .684	.000
Repetition	**Repetition**	*r* = .680	.000
LATA total	**QAB‐TR total**	*r* = .812	.000

The Spearman correlation analysis between the QAB‐TR subsection scores and the total QAB‐TR score (0.244–0.897) revealed statistically significant low‐, medium‐, and high‐level relationships (Table 5).

**TABLE 5:** Correlation analysis results regarding Turkish version of QAB (QAB‐TR) subsections and total score.

	1	2	3	4	5	6	7	8
1. Word comprehension	1							
2. Sentence comprehension	0.585^a^	1						
3. Word finding	0.692^a^	0.580^a^	1					
4. Grammatical construction	0.680^a^	0.592^a^	0.754^a^	1				
5. Speech motor programming	0.280^a^	0.297^a^	0.277^a^	0.289^a^	1			
6. Repetition	0.656^a^	0.581^a^	0.654^a^	0.741^a^	0.244^a^	1		
7. Reading	0.671^a^	0.563^a^	0.700^a^	0.753^a^	0.308^a^	0.680^a^	1	
8. QAB‐TR total	0.785^a^	0.897^a^	0.761^a^	0.795^a^	0.375^a^	0.718^a^	0.732^a^	1

^a^ *p* < .01.

The findings of the analysis conducted to determine whether there was a difference between the LATA and QAB‐TR measurements according to the participant groups are given in Table 4. Statistically significant differences in the total and subsection scores of LATA and QAB‐TR were found according to the groups (*p* < .05) (Table 6).

**TABLE 6:** Comparison results of language assessment test for aphasia (LATA) and Turkish version of QAB (QAB‐TR) measurements between participant groups.

	Group	*n*	Mean	Standard deviation	Median	*X* ^2^/*F*	*p*	difference
Speech fluency	Normal^1^	98	31.51	0.933	126.02	109.366	**<.001**	2 < 1 3 < 1 3 < 2
	Acute stroke patients without aphasia^2^	53	30.28	1.524	86.70			
	Stroke patients with aphasia^3^	37	22.65	7.406	22.19			
Auditory comprehension	Normal^1^	98	63.84	3.207	120.32	80.785	**<.001**	2 < 1 3 < 1 3 < 2
	Acute stroke patients without aphasia^2^	53	60.47	6.157	92.47			
	Stroke patients with aphasia^3^	37	42.16	17.516	29.01			
Repetition	Normal^1^	98	19.72	0.729	116.90	75.431	**<.001**	2 < 1 3 < 1 3 < 2
	Acute stroke patients without aphasia^2^	53	19.11	1.340	93.18			
	Stroke patients with aphasia^3^	37	15.16	5.305	37.05			
Naming	Normal^1^	98	43.38	1.690	123.59	107.703	**<.001**	2 < 1 3 < 1 3 < 2
	Acute stroke patients without aphasia^2^	53	41.64	3.132	90.87			
	Stroke patients with aphasia^3^	37	26.43	11.592	22.65			
Reading	Normal^1^	98	47.65	3.249	124.06	94.237	**<.001**	2 < 1 3 < 1 3 < 2
	Acute stroke patients without aphasia^2^	53	42.87	11.218	89.02			
	Stroke patients with aphasia^3^	37	12.81	16.190	24.07			
Grammar	Normal^1^	98	19.55	1.301	117.22	90.431	**<.001**	2 < 1 3 < 1 3 < 2
	Acute stroke patients without aphasia^2^	53	18.68	2.064	97.41			
	Stroke patients with aphasia^3^	37	11.08	5.751	30.16			
Word actions	Normal^1^	98	19.85	0.581	113.26	88.687	**<.001**	3 < 1 3 < 2
	Acute stroke patients without aphasia^2^	53	19.19	1.688	100.00			
	Stroke patients with aphasia^3^	37	12.57	6.108	36.95			
Writing	Normal^1^	98	39.12	1.923	116.07	72.854	**<.001**	2 < 1 3 < 1 3 < 2
	Acute stroke patients without aphasia^2^	53	33.70	12.676	95.91			
	Stroke patients with aphasia^3^	37	11.16	16.062	35.36			
LATA total	Normal^1^	98	284.65	8.104	128.18	107.826	**<.001**	2 < 1 3 < 1 3 < 2
	Acute stroke patients without aphasia^2^	53	265.94	27.275	83.65			
	Stroke patients with aphasia^3^	37	154.03	65.685	20.84			
Word comprehension	Normal^1^	98	9.57	0.864	123.98	100.756	**<.001**	2 < 1 3 < 1 3 < 2
	Acute stroke patients without aphasia^2^	53	8.54	1.494	88.87			
	Stroke patients with aphasia^3^	37	4.57	2.545	24.49			
Sentence comprehension	Normal^1^	98	7.44	2.455	118.56	62.092^a^	**<.001**	2 < 1 3 < 1 3 < 2
	Acute stroke patients without aphasia^2^	53	6.03	2.373	92.60			
	Stroke patients with aphasia^3^	37	2.32	2.192	33.50			
Word finding	Normal^1^	98	9.81	0.407	122.43	110.327	**<.001**	2 < 1 3 < 1 3 < 2
	Acute stroke patients without aphasia^2^	53	9.37	0.843	94.64			
	Stroke patients with aphasia^3^	37	5.22	2.321	20.31			
Grammatical construction	Normal^1^	98	9.71	0.683	126.29	109.445	**<.001**	2 < 1 3 < 1 3 < 2
	Acute stroke patients without aphasia^2^	53	9.05	0.912	87.11			
	Stroke patients with aphasia^3^	37	5.03	2.164	20.89			
Speech motor programming	Normal^1^	98	9.51	1.708	98.74	29.821	**<.001**	3 < 1 3 < 2
	Acute stroke patients without aphasia^2^	53	9.90	0.481	105.16			
	Stroke patients with aphasia^3^	37	8.24	2.626	67.99			
Repetition	Normal^1^	98	9.76	0.582	121.03	83.404	**<.001**	2 < 1 3 < 1 3 < 2
	Acute stroke patients without aphasia^2^	53	9.30	0.824	87.92			
	Stroke patients with aphasia^3^	37	6.98	2.700	33.66			
Reading	Normal^1^	98	9.69	0.760	119.76	98.019	**<.001**	2 < 1 3 < 1 3 < 2
	Acute stroke patients without aphasia^2^	53	8.76	2.635	96.32			
	Stroke patients with aphasia^3^	37	2.39	3.501	24.99			
QAB‐TR total	Normal^1^	98	9.29	0.616	126.24	103.734	**<.001**	2 < 1 3 < 1 3 < 2
	Acute stroke patients without aphasia^2^	53	8.59	0.886	87.77			
	Stroke patients with aphasia^3^	37	4.81	1.944	20.05			

^a^ *F* test.

^1^ Normal.

^2^ Acute stroke patients without aphasia.

^3^ Stroke patients with aphasia.

There were no statistically significant differences (*p* = .538) between the total scores of the three different forms of QAB‐TR (Table 7). The results of the Spearman correlation tests between the measurements revealed that there were highly significant positive correlations between the QAB‐TR Form 1 and QAB‐TR Form 2 total scores (*r* = .997), the QAB‐TR 1 and QAB‐TR Form 3 total scores (*r* = .984), and the QAB‐TR Form 2 and QAB‐TR Form 3 total scores (*r* = .987) (Table 7).

**TABLE 7:** Difference analysis results for Turkish version of QAB (QAB‐TR) Form 1, QAB‐TR Form 2, and QAB‐TR Form 3 measurements.

	*n*	Mean	Standard deviation	Median	*χ* ^2^	*p*
**QAB‐TR Form 1 total**	10	9.63	0.488	2.20	1.238	.538
**QAB‐TR Form 2 total**	10	9.62	0.507	1.95		
**QAB‐TR Form 3 total**	10	9.58	0.470	1.85		

**FIGURE 3:**
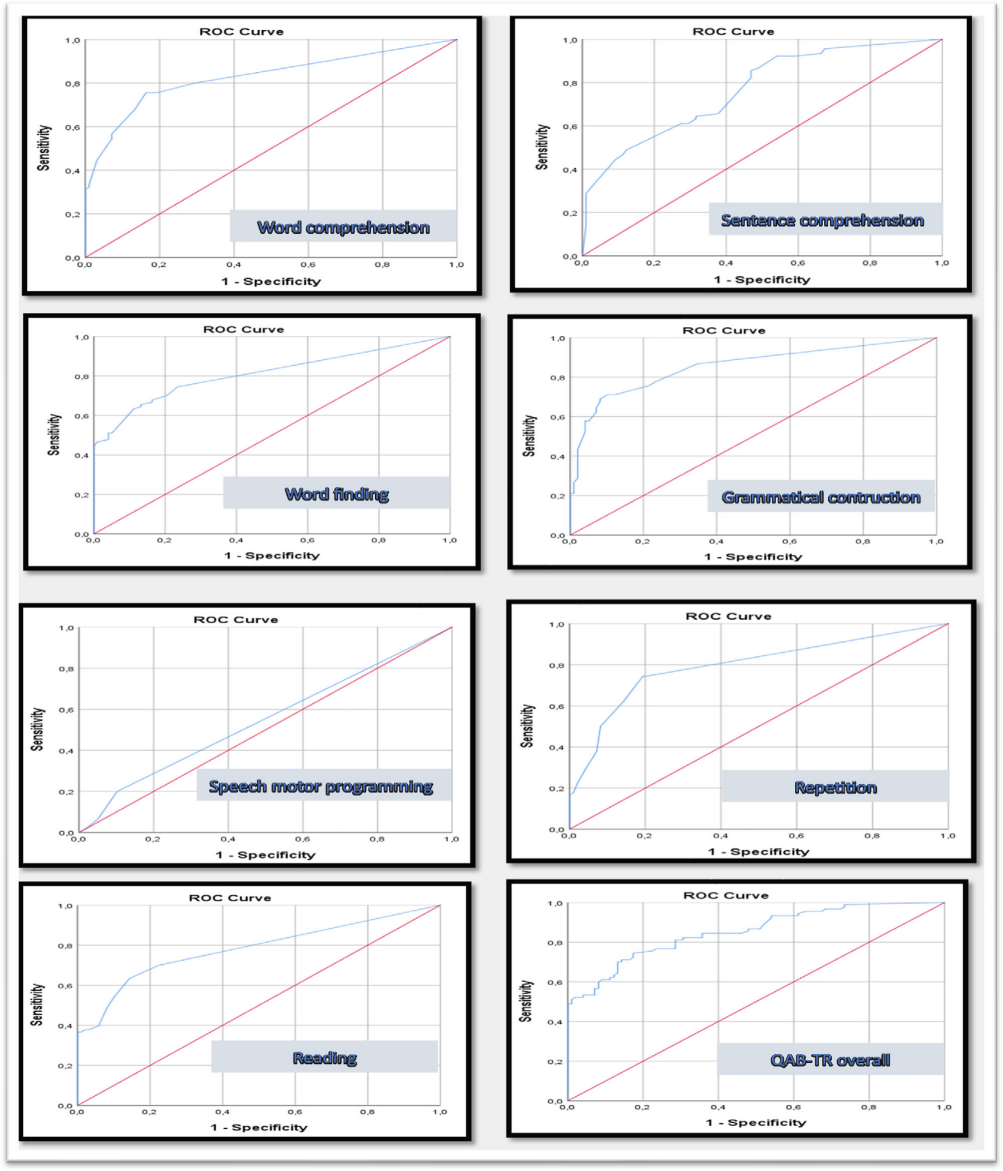
Receiver operating characteristic (ROC) analysis results for Turkish version of QAB (QAB‐TR) subsections and total.

The ROC analysis curve is shown in Figure 3. The area under the ROC curve (AUC) and cut‐off point findings are given in Table 6, and the coordinates related to the curve are given in Figure 3. The AUC was statistically significant and was found to be 0.853 (95% confidence interval: 0.799–0.906). The cut‐off point for the QAB score to discriminate between patients with aphasia and those without aphasia was determined by examining the sensitivity and selectivity values. The cut‐off point was found to be 8.825, with 0.767 sensitivity and 0.765 selectivity (1–0.235) (Table 8).

**TABLE 8:** Turkish version of QAB (QAB‐TR) total and subdomain area under curve (AUC) results.

Variables	Area under curve (AUC)					Cutoff	Sensitivity	Selectivity
	AUC	Standard error	95% CI		*p*			
			Lower limit	Upper limit				
**Word comprehension**	0.828	0.031	0.766	0.889	**<.001**	8.9600	0.756	0.837
**Sentence comprehension**	0.767	0.034	0.701	0.834	**<.001**	6.1250	0.644	0.684
**Word finding**	0.810	0.033	0.746	0.874	**<.001**	9.9150	0.744	0.765
**Grammatical construction**	0.853	0.029	0.797	0.909	**<.001**	9.6900	0.778	0.765
**Speech motor programming**	0.547	0.042	0.464	0.630	.264	8.750	0.200	0.898
**Repetition**	0.795	0.034	0.729	0.861	**<.001**	9.6300	0.744	0.806
**Reading**	0.781	0.035	0.713	0.849	**<.001**	9.7900	0.700	0.776
**QAB‐TR total**	0.853	0.027	0.799	0.906	**<.001**	8.825	0.767	0.765

## 4 DISCUSSION

This study was the first to create a QAB‐TR developed by Wilson et al. (2018) and to test the Turkish version's validity and reliability in PWA. The study was conducted with 188 individuals, including individuals with acute aphasia, chronic aphasia, and acute stroke. The results of the stroke patients without acute aphasia and those of the healthy individuals were also compared. The short QAB‐TR provided an advantage, especially for acute‐phase stroke bedside patients and for patients who would not be able to cooperate during the long assessment period.

In the present study, we compared the QAB‐TR and LATA subdomains that we thought might measure similar features. All the QAB‐TR subdomains except motor speech were found to be associated with LATA. According to the results of the analysis, the scores for QAB‐TR's grammar and word‐finding sections and the QAB‐TR total score were highly correlated when compared to the related subsection of the LATA. In addition, the word comprehension, sentence comprehension, and reading sections were moderately correlated with the corresponding subsections in LATA. These findings indicate that QAB‐TR performs language assessments similar to a test, such as LATA, which conducts a differentiated assessment and has proven validity and reliability, and that it has criterion validity. However, as LATA has no motor speech assessment section, the criterion validity of QAB‐TR's motor speech section cannot be determined. Nonetheless, the very high concordance between the total QAB‐TR and LATA scores supported criterion validity.

QAB‐TR can discriminate between PWA and those without aphasia. In a study conducted by Toğram and Maviş (2012), a statistically significant difference was found between the aphasic and healthy groups across the LATA subsection scores. Similarly, in the present study, a statistically significant difference was found between the total scores of the aphasic and healthy participants (*p* < .001). It was also observed that all the QAB‐TR subscore scores of individuals who had acute stroke differed from those of healthy individuals, which constituted the third group in the present study. This shows that stroke may affect the language functions of individuals in the acute period, albeit to a lesser extent. In the present study, significant differences were found between the scores of the stroke survivors without aphasia and those with aphasia in all the QAB‐TR subsections, except motor speech. These results show that QAB‐TR can discriminate aphasia, even if the language function is affected. The fact that there was no difference in motor speech between stroke PWA and those without aphasia in our study may be due to the fact that most of the PWA who participated in our study did not have dysarthria or apraxia. Wilson et al. (2018) found similar results in the motor speech section in individuals with acute‐period aphasia and who had aphasic stroke. However, it should be noted that the motor speech scores of the stroke patients with aphasia and those without aphasia were lower than those of the healthy participants. This may be because stroke, especially in the acute phase, usually affects the motor components of speech, albeit slightly.

The cut‐off score for QAB in the original study was 8.9 (Wilson et al., 2018), whereas the cut‐off score for QAB‐TR in the present study was 8.825. There is very little difference compared to the English QAB. This may be due to the low use of the passive voice in Turkish, especially in sentence comprehension. A careful examination of the findings revealed that, even in healthy individuals, the average sentence comprehension score was 7.44 out of 10. For this reason, the questions in the sentence comprehension section written in the passive voice, in particular, might have lowered the cut‐off score.

When the correlations of the QAB subitems with each other and with the total score were examined to assess internal consistency, it was seen that there were significant positive correlations between the subsections. The lowest correlation was between motor speech and repetition (low level; *r* = .244), and the highest correlation was between grammar and word finding (high level; *r* = .754). Here, attention and procedural memory are necessary for repetition. Motor speech requires the planning and coordination of motor coordinates and appropriate muscle strength (Duffy, 2019). The fact that motor speech and repetition had the lowest correlation may be due to the very different mechanisms required for their measurement. The high correlation between the subsections in terms of finding directly related nominal words and grammar is in line with the expectations. Significant positive correlations were found between the QAB‐TR subsection scores and the QAB‐TR total score, with the lowest correlation being that between the QAB‐TR total score and the motor speech score (medium level; *r* = .375) and the highest being that between the total score and the sentence comprehension score (high level; *r* = .897). All the findings are positive, and the presence of significant correlations indicates that the scale has internal consistency and that the different subsections assess different areas of language.

The test–retest reliability values of the QAB‐TR total scores did not differ statistically after 2 weeks. The two measures were positively correlated at a high level (*r* = .843). In the retest assessment, a significant difference was found only in the reading section of the QAB‐TR subsections. This may be due to the fact that some people received 3 points for delaying their answers in the first reading and 4 points for reading quickly in the second reading. However, the fact that the other assessment areas and the QAB‐TR total did not differ in the second measurement showed that the test had no learning effect and that the scale had high test–retest reliability. With regard to inter‐rater reliability, the Krippendorff's alpha value in the present study was 0.6754. In the analysis findings, < 0.67 indicates poor agreement, 0.67–0.80 indicates moderate agreement, and ≥80 indicates high agreement. As a result of the analysis, it was determined that there was moderate agreement (alpha = .6754) between the two raters (Krippendorff, 1995). The reason for this may have been the scores given by the undergraduate student SLP because professional experience may affect interpretation. However, the results show inter‐rater reliability.

The sensitivity and selectivity values were 0.767 and 0.765 (1–0.235), respectively. A high sensitivity value indicates the power of the test to identify the patient (Swift et al., 2020). The QAB‐TR sensitivity finding for PWA was found to be high. Specificity is the rate of obtaining a healthy assessment in the diagnostic test among actually healthy conditions. A high specificity rate indicates the power of the diagnostic test to measure healthy conditions as healthy (Swift et al., 2020). Our findings reflect the high probability of a correct diagnosis of QAB‐TR. The AUC (0.853) was statistically significant. An AUC value between 0.8 and 0.9 indicates very good test quality (Metz, 1978). According to our results, the test quality of QAB‐TR is very good.

The fact that the three forms of QAB‐TR are statistically significantly and highly correlated with each other and that their results do not differ statistically indicates that the three forms are consistent with each other and can all be used.

## 5 CONCLUSION

All the study results show that QAB‐TR has internal consistency, criterion validity, test–retest reliability, and inter‐rater reliability. It can be administered in as little as 15 min and provides information about the multidimensional linguistic profiles of individuals. QAB‐TR can be used for both clinical and study purposes as a language battery that allows for the measurement of the strengths and weaknesses of Turkish‐speaking individuals who have suffered a stroke in basic language areas in acute and chronic periods. It can be easily administered at the bedside for individuals who have just suffered acute stroke and can facilitate early assessment of individuals in terms of aphasia and early initiation of therapy, if necessary.

## AUTHOR CONTRIBUTIONS

*Conceptualization; methodology; software; data curation; supervision; resources; formal analysis; visualization; validation; writing—review and editing; writing—original draft; investigation*: Mümüne Merve Parlak. *Conceptualization; methodology; software; writing—review and editing; writing—original draft; formal analysis*: Ayşen Köse.

## CONFLICT OF INTEREST STATEMENT

The authors have no conflicts of interest to declare.

### PEER REVIEW

The peer review history for this article is available at https://publons.com/publon/10.1002/brb3.3343

## Data Availability

All data generated or analyzed during this study are included in this article. Further enquiries can be directed to the corresponding author.
